# Comparison of two nanomaterial labels for detection of SARS-CoV-2 nucleocapsid antigen to improve analytical performance of lateral flow immunoassays

**DOI:** 10.1039/d5ra08197a

**Published:** 2025-12-19

**Authors:** Balaji Srinivasan, Melissa Rivera, Cristina Caicedo, Washington B. Cárdenas, Julia L. Finkelstein, David Erickson, Saurabh Mehta

**Affiliations:** a Cornell Joan Klein Jacobs Center for Precision Nutrition and Health Ithaca NY USA smehta@cornell.edu de54@cornell.edu; b Division of Nutritional Sciences, Cornell University Ithaca NY 14853 USA; c Escuela Superior Politécnica del Litoral (ESPOL) Guayaquil Guayas 090902 Ecuador; d Sibley School of Mechanical and Aerospace Engineering, Cornell University Ithaca NY USA; e Department of Population Health Sciences, Weill Cornell Medicine New York NY USA

## Abstract

Enhancing the sensitivity and detection limits of lateral flow immunoassays (LFAs) remains critical for broadening their diagnostic value and uptake, particularly given the reduced performance of some SARS-CoV-2 antigen tests at low viral load concentrations. The choice of a detection label such as the widely used 40 nm gold nanoparticles (AuNPs), strongly influences LFA analytical performance. Cellulose nanobeads (CNBs), recently introduced in several colors and diameters ranging from 330–365 nm, represent an emerging alternative, though comprehensive performance data are still limited. In this study, we assessed LFAs incorporating either CNBs or AuNPs, using identical antibody pairs for SARS-CoV-2 antigen detection. By analyzing spiked samples and archived nasopharyngeal specimens characterized by RT-qPCR, we examined how viral load and cycle threshold values correlated with antigen test positivity across the two label types. The visual cut-off limit concentration of CNB-based LFAs was observed to be two orders of magnitude lower than the WHO target product profile (TPP) benchmark of 10^6^ copies per mL, whereas AuNP-based LFAs met the defined TPP threshold. Both CNB- and AuNP-LFA formats also met the WHO TPP acceptable targets for time to result, number of user steps and target use setting. These results indicate that CNBs can provide superior analytical sensitivity relative to 40 nm AuNPs and may support more sensitive, visually interpreted LFAs without the need for specialized test strip readers.

## Abstract 2

Schematic of a COVID-19 Ag LFA configuration consisting of a sample pad, conjugate pad, nitrocellulose membrane, and absorbent pad using Au or CNB as a label.

## Graphical abstract

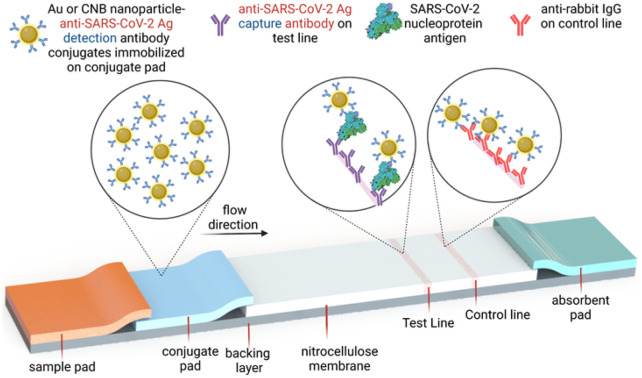

## Introduction

Lateral flow assay (LFA)^1^ is a widely applied, easy-to-perform, and cost-effective analytical technique suitable for the screening of a variety of diseases,^2,3^ environmental monitoring,^4^ food analysis,^5,6^ and detection of a range of analytes^7–9^ in various types of settings.^10,11^ LFAs are a key component of any strategy to expand low cost access to diagnostics particularly at the point-of-care given that most of the world's population cannot afford or access screening or diagnostic tests.^12^ A key challenge for LFAs is their limit of detection that can prevent them from wider clinical uptake and use in population level programs. In this manuscript, we demonstrate ways of improving the analytical performance of LFAs using SARS-CoV-2 as an example, and conduct a small scale diagnostic test accuracy study against RT-PCR in spiked samples as well as archived clinical samples.

### Lateral flow assays and setup

The detection label^13^ is one of the critical components of LFA that provides the signal intensity per binding event and can affect the limit of detection and overall analytical performance. Spherical gold nanoparticles (AuNP),^14^ typically 40 nm in diameter, are the most commonly used label for LFAs due to well-established gold-antibody conjugation protocol in both covalent and passive conjugation approaches, long term stability proven by multiple LFAs reported over the years. In order to improve the assay sensitivity and lower limit of detection, numerous other labels for LFA have been developed – colored particles such as gold nanoshells,^15^ gold nanoflower,^16^ latex microparticles (LNP),^17^ carbon nanoparticles,^18^ fluorescent particles^19^ such as up-converting phosphor nanoparticles (UPN),^20^ europium nanoparticles (EuNP),^21^ Raman probes^22^ and magnetic nanoparticles (MNP).^23^ The detection label properties to be considered during selection for a LFA application include excellent colloidal and storage stability, conjugation efficiency with recognition elements such as an antibody without altering the molecule activity and functionality, reduced non-specific binding to prevent false positive signals, and low cost. More recently cellulose nanobeads (CNBs) became commercially available and are specifically developed for LFAs in red, blue, black, and green colors with average diameters ranging from 330–365 nm. Preliminary data from the supplier indicates that CNB can provide ∼10× lower limit of detection based on performance results of LFA for human chorionic gonadotropin (hCG), troponin and influenza virus detection in clinical samples. The performance of CNBs for antigen-based LFA for seasonal influenza was reported for distinguishing influenza A and B viruses from clinical samples^24^ and performance was compared with gold and latex-labeled LFAs. Similarly, CNB-labeled LFA for rapid detection of SARS-CoV-2 spike-antigens has also been reported.^25^ These results show that CNB-labeled LFA has the potential to improve the performance of LFAs. However, the performance data on CNB-labeled LFA is currently limited and requires further investigation.

### COVID-19

At the end of 2019, Coronavirus disease 2019 (COVID-19),^26,27^ an infectious disease caused by severe acute respiratory syndrome coronavirus 2 (SARS-CoV-2)^28,29^ spread worldwide rapidly. In February 2020, COVID-19 was declared a global health emergency by the World Health Organization (WHO). Early diagnosis and isolation are critical for preventing individual level morbidity and mortality as well as reducing transmission of SARS-CoV-2 virus infection. The two major types of initial diagnostic testing recommended for COVID-19 include: nucleic acid amplification test (NAAT), more commonly a reverse-transcription polymerase chain reaction (RT-PCR) assay, and an antigen test, more commonly a rapid test strip kit designed for self-testing in home settings.

NAATs detect SARS-CoV-2 RNA and are typically performed in laboratory settings. Numerous RT-PCR^30–32^ assays are available around the world with different assays amplifying and detecting different regions of the SARS-CoV-2 genome. Other NAATs^33^ for COVID-19 that are not as widely used include isothermal amplification,^34^ clustered regularly interspaced short palindromic repeats (CRISPR)-based assays,^35–37^ and next-generation sequencing.^38,39^

Antigen assays capable of swiftly detecting SARS-CoV-2 antigens can be conducted promptly and at point of care (POC), are easily accessible and have shorter time-to-result (typically 10–15 minutes) compared to the majority of NAATs. Many home antigen test kits^40^ for SARC-CoV-2 detection are now commercially available and are designed to allow individuals to test themselves with self-collected nasal swabs, and therefore eliminate the need to visit a testing site or clinic. Antigen tests typically have less diagnostic sensitivity than NAATs but are suitable for initial screening where NAATs are not easily accessible or unavailable or when turnaround times with NAAT are too long. Although antigen testing cannot detect SARS-CoV-2 at low concentrations as NAAT can, its sensitivity is highest during the first week of symptoms when viral load is at its highest.

## Materials and methods

### Reagents and materials

Cellulose nanobeads CNBs (NanoAct™, Asahi Kasei Corp, Japan) of type RE1AA (red, 330 nm diameter), Gold Conjugation Kit (ab154873, Abcam) (40 nm, 20 OD), 1% casein blocking buffer, blocking solution (cat# 110050, Boca Scientific), Tris buffer (pH 7.0), 50 mM borate buffer (pH 10.0), Tween-20, conjugate pad (PET coarse Fiber, Type 250Y) (0.54 mm thick), monoclonal anti-SARS-CoV-2 nucleoprotein (3CV4 NP3706, HyTest), monoclonal anti-SARS-CoV-2 nucleoprotein (3CV4 C524, HyTest), purified SARS-CoV-2 nucleoprotein (8COV3, HyTest). Conjugate buffer was sourced from Scripps Laboratories. Secondary anti-Rabbit-IgG (cat# 111-005-144) antibody was sourced from Jackson ImmunoResearch. Bovine serum albumin (BSA), phosphate buffer saline (PBS) buffer, MES buffer, and Tween-20, were sourced from Sigma-Aldrich. Conjugate pad (cat# 8980, 10 mm wide), sample pad (cat# 8951, 10 mm wide), absorbent pad (cat# 440, 20 mm wide) was acquired from Ahlstrom-Munksjö. Nitrocellulose membrane (grade CN-140) was sourced from Sartorius. Backing card and cassettes were sourced from DCN Dx.

### Equipment

Ultrasonic cleaner (DK-300s), tube rotator (Roto-Therm™ model H2024), centrifuge (Eppendorf 5415D), lateral flow reagent dispenser (Matrix 1600, Kinematic Automation), test strip cutting module (Matrix 2360, Kinematic Automation) spectrophotometer (V-1200, VWR).

### Preparation of CNB-anti-SARS-CoV-2-antibody conjugates

A 10% stock solution (60 µL volume) of CNBs was diluted to 1% (600 µL final volume) by adding 540 µL of Tris buffer (pH 7.0). The 1% CNB solution was further mixed with 60 µL of anti-SARS-CoV-2 nucleoprotein antibody (Det-Ab) (1 mg mL^−1^) and incubated for 2 h at 37 °C on the rotomixer. Following incubation, 7.2 mL of 1% casein blocking buffer was added to the CNB-SARS-CoV-2 conjugate solution and incubated for 1 h at 37 °C on the roto-mixer. The blocked conjugates were centrifuged at 13 000*g* for 20 min. The supernatant was discarded, and the pellet was resuspended in 7.2 mL of borate buffer (pH 10.0). Conjugate solution was vortexed briefly and ultrasonicated for 30 s to ensure complete dispersion of CNB-Det-Ab conjugates within the solution. The CNB-Det-Ab conjugate solution was centrifuged at 13 000*g* for 20 min and the pellet was resuspended in 600 µL of conjugate dilution buffer. The CNB-Det-Ab conjugate solution was ultrasonicated for 30 s and the final stock solution at 1% was stored at 4 °C.

### Preparation of AuNP-anti-SARS-CoV-2-antibody conjugates

All the reagents of the AuNP conjugation kit were first brought to room temperature. The anti-SARS-CoV-2 nucleoprotein antibody (Det-Ab) (1 mg mL^−1^) was diluted to 0.1 mg mL^−1^ with the antibody diluent provided in the kit. Diluted antibody (12 µL) was then added to 42 µL of the reaction buffer provided in the kit and 45 µL of this mixture was added to the lyophilized AuNP vial in the kit. The mixture in the vial was gently pipetted to ensure mixing and resuspension of the AuNP and antibody solution and allowed to incubate at room temperature for 15 min. After 15 min of incubation, 5 µL of quencher provided in the kit was added to the vial and the reaction was left at room temperature for 5 min to yield 50 µL of AuNP-Det-Ab conjugate. To remove any unbound antibodies, ten times the volume of the 1 : 10 diluted quencher was added to the conjugate in the vial, followed by centrifugation at 9000*g* for 10 minutes. The supernatant was carefully discarded, and the AuNP-Det-Ab conjugate pellet was resuspended in 1 : 10 diluted quencher in DI water to obtain 20 OD stock solution and stored at 4 °C.

#### Preparation of lateral flow test strip

**Fig. 1:**
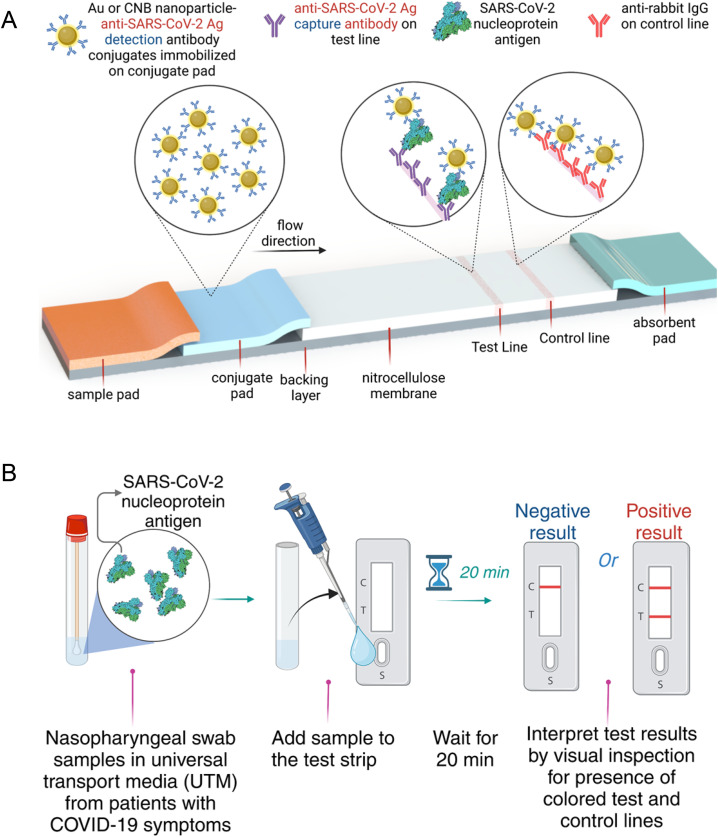
(A) Schematic of LFA configuration consisting of a sample pad, conjugate pad, nitrocellulose membrane, and absorbent pad. The test line on the nitrocellulose membrane contains anti-SARS-CoV-2 antigen capture antibody for detection of the SARS-CoV-2 nucleoprotein antigen. Control line includes the anti-rabbit-IgG antibody. (B) Archived nasopharyngeal swab samples in universal transport media (UTM) from patients with COVID-19 symptoms was added to the test strip. After 15 min, the test result is interpreted by visual inspection for the presence of colored red and test lines.

Fig. 1 shows the lateral flow assay configuration with various components of the test strips and a sandwich format immunoassay for detection of SARS-CoV-2 nucleoprotein. The reagent dispenser (flow rate 10 µL cm^−1^) was set up to dispense monoclonal anti-SARS-CoV-2 capture antibody (Cap-Ab) (1 mg mL^−1^ in PBS buffer) as the test line and secondary anti-mouse IgG (1 mg mL^−1^ in PBS buffer) as the control line. Membranes were dried at 37 °C for 2 h. The nitrocellulose membranes with striped antibodies were further treated with blocking solution for 10 min and dried again in the oven at 37 °C for 2 h. For CNB-LFA strips – the conjugate pad (10 mm wide) was treated with 0.1% tween-20 and dried at 37 °C for 2 h. The nitrocellulose membranes with striped antibodies were further treated with blocking solution for 10 min and dried again in the oven at 37 °C for 2 h. For CNB-LFA strips – the conjugate pad (10 mm wide) was treated with 0.1% Tween-20 and dried at 37 °C for 2 h. The CNB-Det-Ab stock solution was diluted to 0.03% in conjugate buffer (10 mM 2-amino-2-methyl-1-propanol (pH 9.0), 0.5% BSA, 0.5% β-lactose, 0.1% Tween-20, and 0.05% sodium azide) and applied to the treated conjugate pad. The conjugate pad with applied conjugates was dried at 37 °C for 2 h. For AuNP-LFA strips, the AuNP-Det-Ab conjugates were diluted to 0.5 OD in conjugate buffer (PBS 1×, 0.5% BSA, 0.1% Tween-20 and 0.05% sodium azide) and applied to conjugate pad (GFCP103000, 10 mm wide). The conjugate pad with applied conjugates was dried at 37 °C for 2 h. Sample pads (15 mm wide) were treated with blocking buffer and dried at 37 °C for 2 h. The prepared conjugate pads, sample pads and wasted pads were assembled on the backing card. The assembled LFA card was cut into 4.8 mm wide strips using the programmable shear cutter. Individual strips were packaged within plastic cassettes and stored in sealed foil pouches at room temperature.

### Testing protocol

Fig. 1B shows a schematic of the various steps involved in performing the point-of-care testing. Briefly, the user first adds the test sample comprising a mixture of the sample (spiked SARS-CoV-2 buffer or archived nasopharyngeal samples) and running buffer (1× PBS with 1% Tween-20 and 0.1% sodium azide) to the test strip to initiate the flow within the test strip. The SARS-CoV-2 antigen in the test sample reacts with the Det-Ab bound to the label which is stored in the conjugate pad in dry form and flows down the membrane to further interact with the antibodies at the test and control lines immobilized on the membrane. The sample is finally collected in the waste pad. If an antigen is present in the test sample, it is first captured by Det-Ab bound to CNB or AuNP, and this antigen-Det-Ab complex is captured by the Cap-Ab immobilized on the nitrocellulose membrane to form a sandwich complex. After 20 min of sample addition to the test strip, the appearance of red color in the test and control lines is interpreted by observing with naked eye.

### Ethics and use of human samples

All experiments were performed in accordance with the Guidelines of the Ministry of Heath of Ecuador, and experiments were approved by the Ministry of Health emergency COVID-19 IRB “Comité Expedito”, with code 107-2021. Informed consents were obtained from all participants of this study.

We used archived and deidentified nasopharyngeal swab samples at the Laboratory for Biomedical Research, Escuela Superior Politécnica del Litoral (ESPOL). The nasopharyngeal swab samples that were used for the test validation at ESPOL were collected during the COVID-19 emergency through a program to involve Universities with RT-qPCR capacities to help the Ecuadorian Ministry of Health to monitor COVID-19. For universities to be part of the diagnostic program, they were authorized first by an Ecuadorian Ministry of Health Agency called ACESS.

## Results

### Comparison of signal intensities between CNB and AuNP-based LFA with spiked samples

**Fig. 2:**
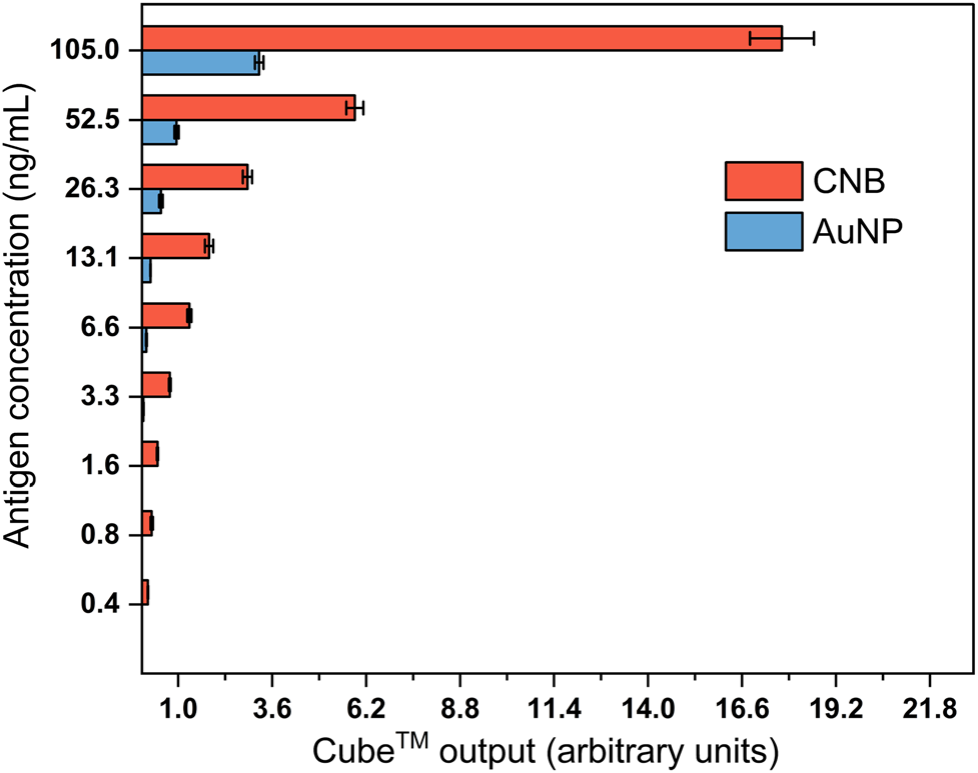
Comparison of cube output signal from CNB- and AuNP-labeled LFA for a range of antigen concentrations.

A series of concentrations of SARS-CoV-2 nucleocapsid protein samples were tested using both CNB and AuNP-based LFA for comparing the test strip signal output measured using the Cube™ reader. Fig. 2 shows a signal comparison plot constructed by plotting Cube™ output measured for a range of nucleocapsid antigen concentrations, where Cube™ output is defined as the ratio of signal intensity of the test line and control line on the test strip, measured using Cube™ after completion of the test. Testing was considered completed 20 min after addition of the test sample to the test strip. Test strips were quantified by the Cube™ reader when the test line was visible to the naked eye. Error bars indicate the standard deviations of output values measured with the Cube™ from spiked samples tested in triplicate. The Cube™ output values were observed to increase with increasing antigen concentration in the test sample- as expected for a sandwich assay format. The lowest concentration that resulted in a visible test line was 3.3 ng mL^−1^ for AuNP-labeled and 0.4 ng mL^−1^ for CNB-labeled test strip. At 3.3 ng mL^−1^, the lowest concentration with visible test line for AuNP-labeled test strip, the Cube™ output for CNB-labeled test strip was ∼20 times higher than AuNP-labeled test strip.

### Testing with archived nasopharyngeal swab samples

**Fig. 3:**
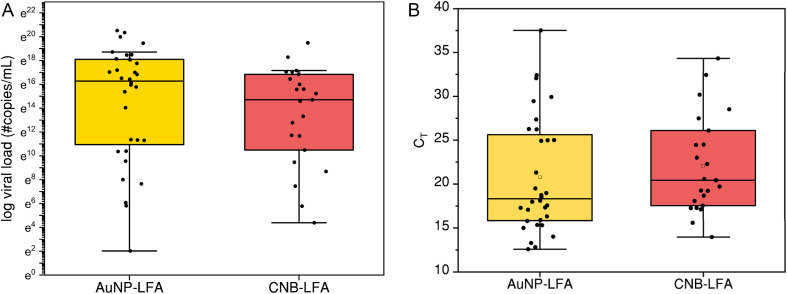
(A) Distribution of viral load (#copies per mL) of the clinical nasopharyngeal swab samples tested with AuNP-LFA and CNB-LFA (B) distribution of *C*_T_ values of the clinical nasopharyngeal swab samples tested with AuNP-LFA and CNB-LFA.

We used archived and deidentified nasopharyngeal swab samples at the Laboratory for Biomedical Research, Escuela Superior Politécnica del Litoral (ESPOL). These samples were previously characterized by gold standard RT-qPCR assay for SARS-CoV-2. Fig. 3 shows the distribution of viral load (copies per mL) and the *C*_T_ values for the various samples that were tested with AuNP-LFA and CNB-LFA. For AuNP-LFA, RT-qPCR negative (*N* = 10) and positive (*N* = 32) samples were randomly selected. For each sample tested, 100 µL of the liquid transport medium sample was added to the sample inlet of the test strip. For CNB-LFA, RT-qPCR negative (*N* = 10) and positive (*N* = 23) samples were randomly selected. For each sample tested, 100 µL of the liquid transport medium sample and 50 µL of running buffer was added to the sample inlet of the test strip.

**Fig. 4:**
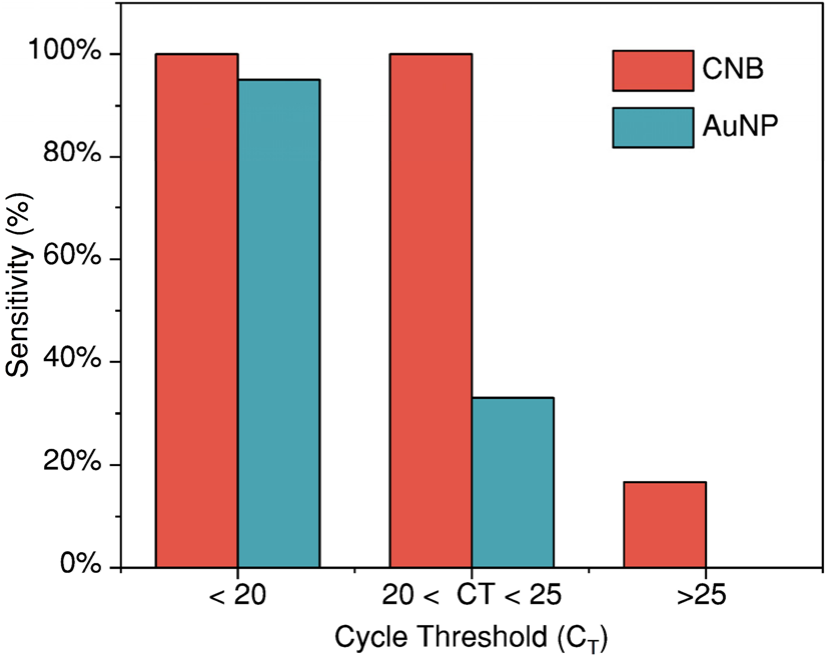
Comparison of sensitivity of CNB and AuNP-LFA with respect to cycle threshold values of the positive SARS-CoV-2 nasopharyngeal swab samples confirmed by RT-qPCR.

Test strips were observed by the naked eye after 20 min to check for the presence of test and control lines to determine if the test was positive/negative. All the RT-qPCR negative samples resulted in negative results with both CNB-LFA and AuNP-LFA with a specificity of 100%. The RT-qPCR positive samples were classified into three ranges (<20, 20–25, >30) based on the *C*_T_ values. Fig. 4 shows the variation of sensitivity for both AuNP-LFA and CNB-LFA with respect to *C*_T_ value distribution. For all positive samples with *C*_T_ > 25, testing with AuNP-LFA yielded a negative result with zero sensitivity. Among the subset of samples tested, the sample with the highest *C*_T_ that showed a positive result with AuNP-LFA was *C*_T_ of 21.31 equivalent to 1.26 × 10^6^ genomic copies per mL. In case of CNB-LFA, the sample with the highest *C*_T_ that showed a positive result with CNB-LFA was *C*_T_ of 26.10 equivalent to 3.59 × 10^4^ genomic copies per mL.

## Discussion

Lateral flow assays (LFAs) were the most used antigen tests for detection of SARS-CoV-2 in nasopharyngeal swab samples. LFAs^41,42^ are membrane-based point-of-care (POC) tests that have been widely used for detection of various target analytes due to their low cost, ease of use and faster results that can be visualized without a need for complex instrumentation. The performance of LFAs depends on the optimal selection of various membrane components, capture and detection antibodies/proteins, various reagents used, strip assembly process and the type of detection labels used. The detection limit of the LFAs for a particular analyte can vary depending on the choice of labels. LFAs based on AuNP are the most widely used over the years due to their long-term stability, low cost of production, well established conjugation chemistry for binding to antibodies, where test results can be interpreted by naked eye inspection without a reader. LFAs based on QDs and EuNP were developed to further reduce the lower detection limits that were previously demonstrated by AuNP labels. However, fluorescent labels such as QDs and EuNP require additional hardware and software to interpret the test results with a test strip analyzer.

The use of CNBs for LFA development has been reported in two studies so far but has not otherwise been widely studied and performance data in comparison with traditional GNP-label is still limited. In LFAs, nanoparticles serve as detection labels and are coupled with biorecognition elements such as antibodies to enable specific binding interaction with the target analyte at the test line. Nanoparticles applied in LFAs are generally larger than the biomolecules attached to them, and the surface area available for each biomolecule (parking area) depends on the particle's surface area, which scales proportionally to the radius squared in the case of spherical particles. CNB particles with a diameter of 330 nm provide increased parking area (∼68 times) for the detection-antibody when compared to AuNP of 40 nm, which potentially increases the chance of antibody-antigen capture. The higher content of the optical signaling moiety in larger diameter nanoparticles can produce stronger signals per binding event and thereby influences the sensitivity of the LFA. Steric hindrance during antibody–antigen binding is also influenced by particle surface curvature, with 330 nm CNB surfaces appearing essentially planar in contrast to the high curvature of 40 nm AuNP at the biomolecule scale. The potential variations in the surface chemistry of CNBs and AuNPs can alter antibody conjugation efficiency, ultimately affecting the sensitivity and effectiveness of the assay. Optimizing particle size and material of nanoparticles is therefore an important consideration for improving assay performance, especially when detecting low-abundance target analytes.

Herein, we developed both CNB and AuNP-labeled LFAs with the same sandwich antibody pairs for detection of SARS-CoV-2 antigen. We contrasted the relationship between *C*_T_ value, viral load, and antigen test positivity to compare the performance of AuNPs and CNBs as labels for rapid antigen test. We first compared the results of detecting SARS-CoV-2 antigen spiked buffer samples with CNB-labeled LFA against the more commonly used 40 nm AuNP-labeled LFA. We observed that the CNB-labeled LFA provided a higher intensity of colorimetric signals measured by the Cube™ reader over the entire range of spiked antigen concentrations (0.41–105 ng mL^−1^) tested. It was also observed that the lowest concentration of antigen at which both test and control lines were visible to naked eye was at 3.28 ng mL^−1^ for AuNP-labeled LFA compared to antigen concentration as low as 0.41 ng mL^−1^ for CNB-labeled LFA. The percentage increase in colorimetric signal intensity with CNB-labeled LFA when compared to AuNP-labeled LFA ranged from 81.69% (at antigen concentration 105 ng mL^−1^) to 95.05% (at antigen concentration 3.28 ng mL^−1^). CNB-labeled LFA was observed to provide ∼8 times lower detection limit than AuNP-labeled LFA when test results were interpreted by naked eye, without the need for any additional signal amplification steps or the need for a dedicated test strip reader.

We then tested AuNP-LFA and CNB-LFA with randomly selected clinical samples that had previously tested positive and negative for SARS-CoV-2 by RT-qPCR. We define the visual cut-off limit concentration as the lowest viral load of the clinical sample that produces a discernible test line when evaluated by visual inspection of the test strip by the user. This threshold reflects the qualitative nature of the assay, which in this study is assessed through visual interpretation rather than reader-based quantification. In contrast to a statistically derived diagnostic limit of detection that requires serial dilutions and multiple replicates, the visual cut-off reported here represents the minimum viral load at which a positive result can be visually identified and is therefore appropriate for characterizing the performance of qualitative, visually interpreted assays. The WHO target product profile (TPP)^43^ for COVID-19 states that the acceptable analytical limit of detection (LOD) is 10^6^ copies per mL. We observed that the AuNP-LFA (visual cut-off limit concentration 1.26 × 10^6^ copies per mL) met the acceptable TPP and CNB-LFA (visual cut-off limit concentration 3.59 × 10^4^ copies per mL) exceeded the acceptable TPP by 2 orders of magnitude. Moreover, both AuNP-LFA and CNB-LFA meet the WHO TPP acceptable targets for time to result, number of user steps and target use setting. We demonstrated that LFA types described here were used by trained staff at ESPOL, therefore meeting the WHO desirable criteria for target use settings and end user profile in community level health centers. The staff at ESPOL were remotely trained over a video call within 30 minutes and met the WHO desirable criteria for training need of “2 hours with instructions for use and quick reference guide”.^43^

Detection labels are a critical component of the overall analytical performance of LFA applications. Labels such as AuNP, LNP and CNB can be used for both applications that do not necessarily require a test strip reader to provide test results. Labels such as EuNP and QDs require an excitation light source and require additional optics or readers for interpreting test results. For rapid testing in self- and home testing scenarios for applications where positive or negative result interpretation would suffice, eliminating the need for a dedicated reader can provide cost benefit. In retrospective studies of study population that tested positive for COVID-19 post-vaccination, results suggest^44^ that two-dose vaccination reduced viral load with accelerated viral clearance in patients. Similarly, post-booster dose cases were shown in another study^45^ to be associated with a significantly higher median *C*_T_ value (lower viral load) than cases in unvaccinated individuals. In populations with previous multiple-dose vaccinations, the lower limit of detection of CNB- over AuNP LFA for viral load by two orders of magnitude for COVID-19 antigen test will enable rapid tests to detect even lower viral loads without the need for a reader. The availability of CNB in multiple colors has the potential for application in multiplexed color-coded lateral flow assays to achieve lower limits of detection than conventional gold nanoparticle label, with options of both quantitative and qualitative interpretation of results with/without the need for a dedicated test strip reader.

### Strengths and limitations

The sample sizes used for comparing the CNB-LFA and AuNP-LFA tests with clinical specimens were not identical, resulting in a non-paired comparison. This discrepancy was primarily due to limited sample availability at the time of the study and can be addressed in future investigations with a larger and paired sample set. The focus of this study was on developing a proof-of-concept prototype and evaluating its performance using spiked and archived clinical samples, while keeping early-stage development costs low. Additional studies are needed to increase the sample size and enable a more comprehensive adequately-powered evaluation of the CNB-LFA's diagnostic performance including determination of its LOD.

## Conclusions

In summary, we developed CNB and AuNP-labeled LFAs with the same sandwich antibody pairs for detection of SARS-CoV-2 antigen. Both CNB and AuNP-based LFA test strips were tested in parallel with archived nasopharyngeal swab samples that were previously characterized by RT-qPCR as gold standard reference method. We compared the relationship between *C*_T_ value, viral load, and antigen test positivity to compare the performance of AuNPs and CNBs as labels for rapid antigen test. Results indicate the visual cut-off limit concentration of AuNP-LFA met the acceptable WHO TPP for COVID-19 and CNB-LFA exceeded the acceptable TPP by 2 orders of magnitude. Both CNB- and AuNP LFA meet the WHO TPP acceptable targets for time to result, number of user steps and target use setting. Our preliminary results here indicate the application of CNB for lateral flow assay development will have an impact on performance improvement for various other assays.

## Author contributions

Conceptualization – SM, DE, WBS, JLF, BS; data curation – MR, CC, BS; formal analysis – MR, CC, BS; funding acquisition – SM, DE, WBS, JLF; investigation – MR, CC, BS; methodology – MR, CC, BS, WBS, SM; software – MR, CC, BS; supervision – SM, DE, WBS, JLF; visualization – BS; writing original draft – BS; writing review and editing – BS, MR, CC, SM, DE, WBS, JLF; all authors read and approved the submitted version of the manuscript.

## Conflicts of interest

The authors declare that they have no known competing financial interests or personal relationships that could have appeared to influence the work reported in this paper.

## Abbreviations

LFA: Lateral flow assay
OD: Optical density
AuNP: Gold nanoparticle
CNB: Cellulose nanobeads
NP: Nucleocapsid protein
*C*_T_
: Cycle threshold
RT-qPCR: Reverse transcription-quantitative polymerase chain reaction
LOD: Limit of detection
POC: Point of care

## Data Availability

The data generated to compare the developed assay against the reference standard is included in the results and figures in the manuscript.
